# Morphological and genetic divergence in Swedish postglacial stickleback (*Pungitius pungitius*) populations

**DOI:** 10.1186/1471-2148-11-287

**Published:** 2011-10-04

**Authors:** Kenyon B Mobley, Daniel Lussetti, Frank Johansson, Göran Englund, Folmer Bokma

**Affiliations:** 1 Department of Ecology and Environmental Science, Linnaeus väg 6, Umeå University, 90187 Sweden

## Abstract

**Background:**

An important objective of evolutionary biology is to understand the processes that govern phenotypic variation in natural populations. We assessed patterns of morphological and genetic divergence among coastal and inland lake populations of nine-spined stickleback in northern Sweden. Coastal populations are either from the Baltic coast (n = 5) or from nearby coastal lakes (n = 3) that became isolated from the Baltic Sea (< 100 years before present, ybp). Inland populations are from freshwater lakes that became isolated from the Baltic approximately 10,000 ybp; either single species lakes without predators (n = 5), or lakes with a recent history of predation (n = 5) from stocking of salmonid predators (~50 ybp).

**Results:**

Coastal populations showed little variation in 11 morphological traits and had longer spines per unit of body length than inland populations. Inland populations were larger, on average, and showed greater morphological variation than coastal populations. A principal component analysis (PCA) across all populations revealed two major morphological axes related to spine length (PC1, 47.7% variation) and body size (PC2, 32.9% variation). Analysis of PCA scores showed marked similarity in coastal (Baltic coast and coastal lake) populations. PCA scores indicate that inland populations with predators have higher within-group variance in spine length and lower within-group variance in body size than inland populations without predators. Estimates of within-group *P*_ST _(a proxy for *Q*_ST_) from PCA scores are similar to estimates of *F*_ST _for coastal lake populations but *P*_ST _>*F*_ST _for Baltic coast populations. *P*_ST _>*F*_ST _for PC1 and PC2 for inland predator and inland no predator populations, with the exception that *P*_ST _<*F*_ST _for body size in inland populations lacking predators.

**Conclusions:**

Baltic coast and coastal lake populations show little morphological and genetic variation within and between groups suggesting that these populations experience similar ecological conditions and that time since isolation of coastal lakes has been insufficient to demonstrate divergent morphology in coastal lake populations. Inland populations, on the other hand, showed much greater morphological and genetic variation characteristic of long periods of isolation. Inland populations from lakes without predators generally have larger body size, and smaller spine length relative to body size, suggesting systematic reduction in spine length. In contrast, inland populations with predators exhibit a wider range of spine lengths relative to body size suggesting that this trait is responding to local predation pressure differently among these populations. Taken together the results suggest that predation plays a role in shaping morphological variation among isolated inland populations. However, we cannot rule out that a causal relationship between predation versus other genetic and environmental influences on phenotypic variation not measured in this study exists, and this warrants further investigation.

## Background

Morphological variation is a ubiquitous phenomenon and it exists at many taxonomic levels such as between species, within species and among individuals in populations. Depending on its relationship to the background environment, morphological variation may be either adaptive or non-adaptive [1,2]. The degree to which the environment, phenotypic plasticity, and the underlying genetic architecture interact may also fundamentally influence morphological variation [3-5]. Moreover, divergent morphology is often associated with speciation events suggesting that phenotypic variation plays a role in the speciation process [6,7]. Thus, a fundamental goal of evolutionary biology is to understand what governs patterns of morphological variation in natural populations.

In this study we explore patterns of morphological divergence and the genetic signature of recent isolation in populations of nine-spined stickleback (*Pungitius pungitius*) in northern Sweden. The nine-spined stickleback is a small euryhaline fish that inhabits a variety of freshwater and brackish environments in the temperate and subarctic regions of the northern hemisphere [8,9]. In Sweden, nine-spined sticklebacks are widely distributed in the Baltic sea (3.5 - 8.8 psu [10]) and inhabit a variety of freshwater ponds, lakes and rivers [9].

Throughout its range, the nine-spined stickleback displays remarkable morphological variation, particularly in isolated freshwater populations where morphology appears to be shaped by a combination of isolation, drift and natural selection [11-13]. For example, sticklebacks from landlocked populations are generally larger than their coastal conspecifics [11]. Also, loss of the pelvic girdle and dorsal, pelvic, and anal spines has been observed in several isolated populations [8,9,11-16], making it an excellent model for studies of parallel evolution.

The armour and spines of sticklebacks (Gasterosteidae) are used primarily as a defence against piscivorous fishes and many populations retain both characters in the presence of these predators [17]. However there may be a cost in maintaining such defences in populations lacking predatory fishes, as reduced armour and spines may aid escape from invertebrate predators [18,19]. If so, reduction or loss of these traits should be favoured in populations where invertebrate predators predominate [16,19,20]. Reversal of the loss of armour has also been shown in sticklebacks, suggesting that these defensive traits may be rapidly regained in populations based on the strength of natural selection experienced [21].

The topology of Scandinavia has been strongly influenced by recent climactic events related to its most recent deglaciation after the last glacial maximum approximately 11-10,000 calibrated years before present (ybp) [22,23]. Prior to this time, the region was completely covered by an ice sheet. Fish populations originating from the Baltic Sea presumably became trapped in lakes and ponds formed by land uplift soon after the ice-melt [24,25]. In northern Sweden, nine-spined sticklebacks occur in both single species lakes and lakes with mixed fish communities [25]. Recently, predatory fish such as brown trout (*Salmo trutta*) and rainbow trout (*Onchorynchus mykiss*) have been introduced in a few lakes for recreational angling purposes, offering an ideal platform to explore the role of predation on morphological variation in nine-spined sticklebacks.

There are several goals to the current study. First, we investigate whether populations of nine-spined sticklebacks show genetic signatures of isolation in inland lakes and ponds isolated since the last glacial retreat. Here we investigate patterns of genetic differentiation, genetic variation, and bottlenecking using nine variable microsatellite loci. The second goal of the study is to investigate morphological divergence between populations that share common ecological conditions. To this end, we group populations according to whether they originate from the Baltic coast, coastal lakes, or inland lakes and ponds with or without predators. Finally, we explore the potential for natural selection to affect morphological evolution by comparing phenotypic divergence with neutral genetic divergence using a *P*_ST _- *F*_ST _approach; an alternative to *Q*_ST _- *F*_ST _based on phenotypic variation in natural populations.

## Results

**Figure 1:**
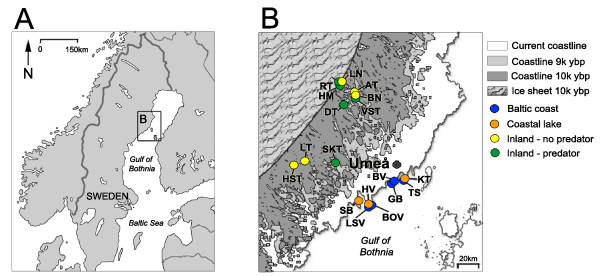
**Study sites in northern Sweden**. A) Location of the study area B) Enlargement of study area and study sites. Current and past coastlines (in calibrated years before present) are shown.

Details concerning the sampling locations and predator regimes can be found in Table 1 and Figure 1.

**Table 1:** Summary of sampled populations of *Pungitius pungitius *included in this study

Site	**Abb**.	n	Lat, Long (N, E)	Group	Community composition^a^
Boggviken	BOV	30	63.46195, 19.73239	Baltic coast	Baltic community
Bölseviken	BV	30	63.66270, 20.21384	Baltic coast	Baltic community
Golfbäcken	GB	30	63.65711, 20.12610	Baltic coast	Baltic community
Laxskärsviken	LSV	30	63.44247, 19.68919	Baltic coast	Baltic community
Tarvsundet	TS	25	63.66600, 20.27221	Baltic coast	Baltic community
Hästviken	HV	29	63.46192, 19.71733	Coastal lake	Crucian carp, perch, pike, roach, three-spined stickleback
Kroktjärn	KT	30	63.68005, 20.39184	Coastal lake	Crucian carp
Storbastugrundet	SB	30	63.50622, 19.44428	Coastal lake	Crucian carp
Abborrtjärn	AT	30	64.47815, 19.43640	Inland - no predator	*na*
Bynästjärnen	BN	30	64.45462, 19.43914	Inland - no predator	*na*
Hornspiggtjärn	HST	30	63.79901, 18.24176	Inland - no predator	*na*
Lapptjärn	LT	30	63.86273, 18.51466	Inland - no predator	*na*
Lill Navartjärn	LN	28	64.56484, 19.19882	Inland - no predator	*na*
Västre Skärträsk	VST	26	64.42704, 19.44671	Inland - predator	Arctic char, brown trout (1969)^b^, burbot, grayling, perch, ruffe, whitefish
Djuptjärnen	DT	30	64.43315, 19.16404	Inland - predator	Arctic char, brown trout (1959, 1968)^b^
Hansmyrtjärn	HM	26	64.55667, 19.17370	Inland - predator	Brown trout
Rörtjärn	RT	30	64.57031, 19.16611	Inland - predator	Brown trout (1985, 1993, 1995, 1999, 2003)^b^
Skrattabbartjärn	SKT	40	63.86905, 19.08938	Inland - predator	Brown trout (1985^b^, 2000^c^), rainbow trout (1985^b^, 2000^c^)

Sites, abbreviations (Abb.), number of individuals sampled from each population (n), coordinates (Lat, Long), group, and fish community composition are listed for each site. *na *= Not applicable.

^a^Data for community composition derived from PIKE database [28]. Species names: arctic char = *Salvenlinus alpinus*, brown trout = *Salmo trutta*, burbot = *Lota lota*, Crucian carp = *Carassius carassius*, grayling = *Thymallus thymallus*, perch = *Perca fluviatilis*, pike = *Esox lucius*, rainbow trout = *Oncorhynchus mykiss*, roach = *Rutilus rutilus*, ruffe = *Gymnocephalus cernuus*, three-spined stickleback = *Gasterosterus aculearus*, whitefish = *Coregonus lavaretus*. ^b^Stocking date. ^c^Last recorded date of presence.

### Genetic analysis

Summary statistics of microsatellite data per population are listed in Table 2. Fisher's exact tests indicated no significant departures from Hardy-Weinberg equilibrium for any locus in any population after application of a Bonferroni adjustment to correct for multiple tests [26]. Tests of genotypic disequilibrium between loci were also non-significant after Bonferroni adjustment, supporting independent assortment of microsatellite loci. Analysis using MICROCHECKER detected possible null alleles at *STN19 *in GB and RT populations and potential null alleles or incorrect scoring due to stutter in *STN196 *in the RT population. Analysis of loci over all populations with LOSITAN suggests that loci are putatively neutral except locus *STN196*, which may be under positive selection, and *STN19*, which may be under balancing selection. We reran the LOSITAN analysis excluding inland populations and these results suggest that all loci in ancestral coastal populations are neutral.

**Table 2:** Summary statistics for microsatellite data arranged by locus

		*Pbbe1125*	*Stn19*	*Stn49*	*Stn96*	*Stn148*	*Stn163*	*Stn173*	*Stn196*	*Stn198*
BOV	*N*	29	30	30	30	30	30	30	30	30
	*A*	7	5	5	6	4	5	7	3	5
	*H*_E_	0.828	0.667	0.500	0.800	0.600	0.633	0.667	0.633	0.600
	*H*_O_	0.709	0.714	0.423	0.673	0.553	0.621	0.629	0.569	0.723
BV	*N*	30	30	30	30	30	30	30	30	30
	*A*	6	3	4	7	3	6	6	5	6
	*H*_E_	0.700	0.633	0.300	0.533	0.467	0.667	0.567	0.667	0.700
	*H*_O_	0.715	0.669	0.299	0.641	0.514	0.624	0.592	0.631	0.706
GB	*N*	28	29	30	30	30	30	29	30	30
	*A*	6	5	4	6	4	6	7	3	6
	*H*_E_	0.607	0.379	0.367	0.567	0.600	0.633	0.690	0.633	0.800
	*H*_O_	0.623	0.670	0.376	0.571	0.525	0.566	0.707	0.532	0.713
LSV	*N*	27	28	30	30	30	30	30	29	29
	*A*	7	4	5	6	4	4	6	4	5
	*H*_E_	0.852	0.821	0.367	0.667	0.433	0.400	0.600	0.655	0.793
	*H*_O_	0.626	0.644	0.329	0.597	0.553	0.346	0.564	0.619	0.685
TS	*N*	24	25	25	22	25	25	24	24	25
	*A*	6	3	4	7	3	4	5	3	5
	*H*_E_	0.625	0.720	0.440	0.682	0.640	0.520	0.542	0.583	0.960
	*H*_O_	0.668	0.650	0.435	0.669	0.509	0.541	0.591	0.611	0.736
HV	*N*	29	29	29	29	29	29	29	29	29
	*A*	7	3	6	5	3	4	6	3	5
	*H*_E_	0.552	0.931	0.448	0.517	0.586	0.414	0.621	0.621	0.655
	*H*_O_	0.710	0.665	0.431	0.569	0.532	0.500	0.598	0.588	0.642
KT	*N*	30	30	30	30	30	30	30	29	30
	*A*	7	3	5	3	3	6	5	3	4
	*H*_E_	0.600	0.900	0.267	0.467	0.500	0.567	0.533	0.655	0.533
	*H*_O_	0.644	0.649	0.302	0.540	0.459	0.595	0.620	0.624	0.534
SB	*N*	30	30	30	30	30	30	30	30	29
	*A*	7	3	5	6	6	7	6	4	6
	*H*_E_	0.733	0.871	0.387	0.581	0.516	0.548	0.645	0.710	0.828
	*H*_O_	0.702	0.676	0.344	0.569	0.594	0.601	0.619	0.620	0.733
AT	*N*	30	30	30	28	30	30	30	30	30
	*A*	3	3	4	3	3	2	4	3	2
	*H*_E_	0.067	0.867	0.833	0.714	0.867	0.233	0.233	0.633	0.433
	*H*_O_	0.066	0.666	0.599	0.571	0.582	0.210	0.273	0.527	0.381
BN	*N*	28	30	30	29	30	30	30	30	30
	*A*	3	3	3	3	2	2	2	3	2
	*H*_E_	0.857	0.833	0.567	0.276	0.567	0.067	0.133	0.333	0.500
	*H*_O_	0.514	0.621	0.493	0.249	0.413	0.066	0.127	0.341	0.381
HST	*N*	29	28	30	30	30	30	30	30	30
	*A*	3	3	2	1	2	2	1	2	1
	*H*_E_	0.621	0.679	0.233	---	0.133	0.767	---	0.100	---
	*H*_O_	0.520	0.547	0.210	---	0.127	0.494	---	0.097	---
LT	*N*	30	29	30	30	30	30	28	27	30
	*A*	4	4	3	1	2	3	3	3	3
	*H*_E_	0.700	0.828	0.100	---	0.733	0.200	0.536	0.333	0.267
	*H*_O_	0.575	0.658	0.098	---	0.472	0.186	0.542	0.352	0.244
LN	*N*	28	28	28	28	28	28	28	28	28
	*A*	2	2	2	3	1	1	3	2	1
	*H*_E_	0.357	0.500	0.036	0.357	---	---	0.286	0.036	---
	*H*_O_	0.299	0.506	0.036	0.309	---	---	0.259	0.036	---
VST	*N*	25	26	26	23	26	26	24	25	26
	*A*	2	2	2	3	2	3	3	3	3
	*H*_E_	0.040	0.462	0.038	0.087	0.154	0.192	0.542	0.120	0.231
	*H*_O_	0.115	0.401	0.038	0.127	0.208	0.212	0.434	0.117	0.278
DT	*N*	29	30	30	30	30	30	30	30	30
	*A*	3	2	2	1	2	1	4	2	2
	*H*_E_	0.517	0.567	0.100	---	0.067	---	0.567	0.167	0.467
	*H*_O_	0.537	0.494	0.097	---	0.066	---	0.524	0.155	0.488
HM	*N*	26	26	26	24	26	26	26	26	26
	*A*	1	2	3	3	2	2	3	2	1
	*H*_E_	---	0.577	0.077	0.625	0.115	0.038	0.192	0.038	---
	*H*_O_	---	0.419	0.076	0.520	0.111	0.038	0.180	0.111	---
RT	*N*	30	30	30	30	30	29	30	30	30
	*A*	2	2	2	3	2	3	2	3	2
	*H*_E_	0.067	0.500	0.067	0.367	0.133	0.552	0.400	0.133	0.033
	*H*_O_	0.066	0.413	0.066	0.635	0.127	0.477	0.488	0.310	0.033
SKT	*N*	39	40	40	40	39	40	40	40	40
	*A*	3	3	1	2	3	2	1	3	3
	*H*_E_	0.641	0.575	---	0.050	0.641	0.075	---	0.200	0.050
	*H*_O_	0.514	0.506	---	0.049	0.483	0.073	---	0.184	0.050

	*A*_TOTAL_	11	5	7	13	9	8	7	7	8

Number of alleles (*A*), sample sizes (*N*), and expected and observed heterozygosity (*H*_E _and *H*_O_) are reported for each population. The total number of unique alleles per locus (*A*_TOTAL_) tallied over all populations is also reported. See Table 1 for description of site abbreviations.

**Figure 2:**
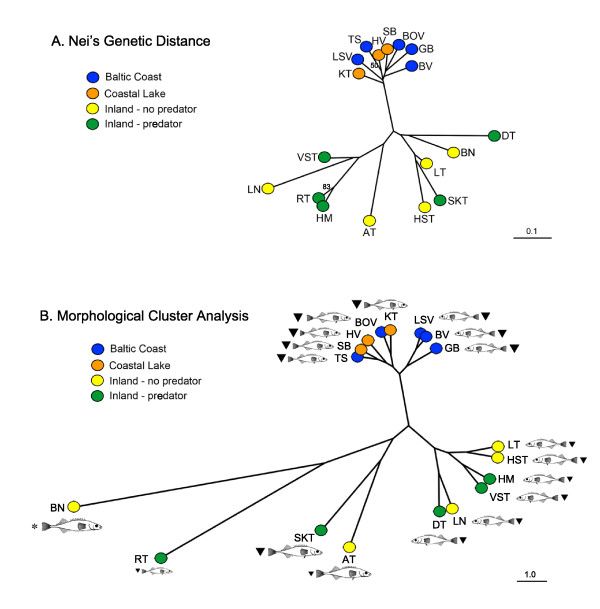
**Genetic and morphological distance measures**. A) Neighbour-joining tree showing the genetic relationships between populations of nine-spined sticklebacks based on Nei's genetic distance (*D*_A_) across all nine microsatellite loci. Percentage of bootstrap support > 50% for 10,000 replicates are shown. B) Cluster analysis based on population means of morphological characters. Mean standard length of fish (fish diagram) and a representative spine length estimate (mean pelvic spine length, upside down triangle) are presented and scaled proportionately for reference. Site abbreviations and groups are defined in Table 1. *The BN population lack pelvic spines.

A neighbour-joining phylogenetic tree using *D*_A _distances based on all microsatellite data demonstrates that Baltic coast and coastal lakes cluster together and are genetically more similar to one another than inland populations with or without predators (Figure 2a). Inland populations showed greater genetic distances, consistent with longer periods of isolation from coastal populations and each other (Figure 2a). Bootstrap support for the branching order of the inland populations is poor, which is in line with our expectation that these populations all originated within a short period of time after the last glacial retreat.

Population pairwise *F*_ST _estimates demonstrate that coastal populations show lower levels of genetic differentiation than inland populations. Among coastal populations, pairwise *F*_ST _values were low (range = -0.005 - 0.026, Table 3) indicating weak population structuring among these populations. In contrast, inland populations had high levels of genetic differentiation as evidenced by high pairwise *F*_ST _values (range = 0.081-0.677). Pairwise standardized *F*_ST _values gave similar results to pairwise *F*_ST _values (Table 3). A nested within-group comparison between Baltic coast and coastal lake populations showed no significant differences in *F*_ST _estimates (P = 1.00). A nested within-group comparison also shows no significant difference between *F*_ST _estimates between inland lakes with or without the presence of predators (P = 0.57). However a significant difference between pooled coastal (coastal lakes, Baltic coast) and pooled inland (predator, no-predator) *F*_ST _estimates is apparent confirming the visual impression from Figure 2 that inland populations show higher levels of within-population genetic differentiation (P = 0.001).

**Table 3:** Population pairwise global *F*_ST _and standardized *F*_ST _estimates for nine microsatellite loci

Group		BOV	TS	BV	GB	LSV	SB	HV	KT	BN	AT	LT	HST	LN	VST	DT	RT	HM	SKT
Baltic coast	BOV	---	-0.005	0.008	0.015	0.036	-0.015	0.008	0.061	0.434	0.441	0.314	0.456	0.581	0.449	0.569	0.486	0.560	0.535
Baltic coast	TS	0.001*	---	-0.001	-0.009	0.001	-0.001	-0.016	0.024	0.362	0.427	0.251	0.393	0.630	0.492	0.513	0.522	0.572	0.503
Baltic coast	BV	0.003*	0.003*	---	0.041	0.041	0.014	0.009	0.017	0.399	0.479	0.300	0.433	0.625	0.431	0.521	0.481	0.560	0.500
Baltic coast	GB	0.006*	0.001*	0.018	---	0.043	0.018	0.011	0.045	0.459	0.472	0.339	0.473	0.612	0.503	0.562	0.574	0.635	0.581
Baltic coast	LSV	0.015*	0.005*	0.019	0.019	---	0.011	-0.014	0.037	0.391	0.420	0.271	0.460	0.579	0.454	0.537	0.482	0.508	0.502
Coastal lake	SB	-0.005*	0.004*	0.007*	0.008	0.006*	---	-0.007	0.044	0.430	0.434	0.302	0.455	0.559	0.440	0.579	0.438	0.502	0.520
Coastal lake	HV	0.003*	-0.003*	0.004*	0.005*	-0.004*	-0.002*	---	0.003	0.398	0.429	0.304	0.461	0.584	0.462	0.536	0.499	0.544	0.525
Coastal lake	KT	0.026	0.014	0.008*	0.021	0.019	0.020	0.001*	---	0.429	0.468	0.345	0.478	0.631	0.497	0.542	0.538	0.591	0.534
Inland - no predator	BN	0.218	0.191	0.205	0.240	0.211	0.220	0.208	0.231	---	0.471	0.393	0.538	0.764	0.579	0.498	0.523	0.534	0.317
Inland - no predator	AT	0.209	0.206	0.234	0.232	0.216	0.210	0.212	0.239	0.289	---	0.490	0.678	0.407	0.507	0.510	0.505	0.564	0.594
Inland - no predator	LT	0.158	0.135	0.154	0.175	0.147	0.156	0.159	0.185	0.248	0.296	---	0.187	0.689	0.476	0.552	0.562	0.617	0.360
Inland - no predator	HST	0.260	0.239	0.253	0.278	0.280	0.265	0.275	0.291	0.378	0.460	0.143	---	0.843	0.709	0.630	0.734	0.780	0.471
Inland - no predator	LN	0.344	0.389	0.381	0.375	0.368	0.338	0.361	0.399	0.557	0.275	0.500	0.677	---	0.513	0.795	0.616	0.709	0.796
Inland - predator	VST	0.241	0.278	0.238	0.280	0.263	0.240	0.259	0.288	0.400	0.330	0.323	0.543	0.387	---	0.613	0.435	0.603	0.627
Inland - predator	DT	0.313	0.301	0.297	0.324	0.321	0.326	0.311	0.323	0.338	0.343	0.381	0.484	0.621	0.454	---	0.777	0.812	0.656
Inland - predator	RT	0.264	0.294	0.267	0.321	0.282	0.243	0.282	0.311	0.352	0.322	0.378	0.545	0.469	0.307	0.557	---	0.098	0.528
Inland - predator	HM	0.328	0.350	0.336	0.385	0.320	0.301	0.333	0.369	0.382	0.384	0.445	0.622	0.581	0.463	0.627	0.081	---	0.548
Inland - predator	SKT	0.321	0.316	0.306	0.358	0.317	0.317	0.326	0.338	0.226	0.417	0.258	0.372	0.641	0.484	0.498	0.399	0.435	---

Standardized *F*_ST _values are above the diagonal, uncorrected *F*_ST _values are below the diagonal. See Table 1 for description of site abbreviations.

Uncorrected *F*_ST _values were bootstrapped over 10,000 replicates and non-significant pairwise differences (α = 0.05) are marked with an asterix.

Inland populations had lower levels of within-population genetic variation than coastal populations. In coastal populations, all nine microsatellite loci were polymorphic, with 3-8 alleles segregating per locus. Baltic coast and coastal lake populations showed similar levels of observed heterozygosity (*H*_o_), allelic richness (*A*), and gene diversity (*h*_s_) (P = 0.88, P = 0.89, P = 0.97 respectively, Table 4). Inland populations, on the other hand, had a maximum of 5 alleles segregating per locus and high levels of fixation ranging from 0-3 loci fixed for a certain allele per population. No two inland populations showed fixation for the same set of loci. Inland populations had significantly lower *H*_o_, *A *and *H*_S _than their coastal counterparts (P = 0.01 for all variables, Table 4) but whether or not an inland population had predators present did not have a significant influence on either *H*_o_, *A*, or *H*_S _(P = 0.31, P = 0.76, P = 0.47 respectively, Table 4).

**Table 4:** Summary statistics for within-group analyses

Group	*N*	*H*_o_	*A*	*h*_s_	*F*_ST_	PC1 *P*_ST _	*c/h*^2 ^	PC2 *P*_ST_	*c/h*^2^
Baltic coast	5	0.615	4.716	0.592	0.010	0.115 (0.048 - 0.198)	0.200	0.097 (0.034 - 0.170)	0.287
Coastal lake	3	0.600	4.517	0.580	0.006	0.062 (-0.013 - 0.153)	*na*	0.027 (-0.013 - 0.126)	*na*
Inland - no predator	5	0.375	2.373	0.303	0.414	0.164 (0.090 - 0.167)	0.283*	0.768 (0.694 - 0.796)	0.312
Inland - predator	5	0.234	2.195	0.227	0.483	0.828 (0.443 - 0.870)	*na*	0.631 (0.477 - 0.699)	*na*

Number of populations (*n*), observed heterozygosity (*H*_O_), allelic richness (*A*), gene diversity (*h*_s_) and *F*_ST _values are listed for each group for all nine microsatellite loci. Within-group *P*_ST _estimates with 95% confidence intervals for PC1 (body size) and PC2 (spine length) and *c/h*^2 ^critical value (assuming *h*^2 ^= 0.4) with conservative 95% confidence limit are also listed. Asterix denotes *h*^2^/*c *critical value for the instance where *P*_ST _<*F*_ST_. *na *= not applicable.

We found evidence of recent population-level bottlenecking in two of the 18 populations investigated. Populations GB and VST both showed a significant heterozygosity deficiency based on our BOTTLENECK model criteria (Wilcoxon sign rank one tailed test: GB, P = 0.0137; VST, P = 0.0137). In all other populations there was no evidence for a departure from mutation-drift equilibrium implying that recent genetic bottlenecking is unlikely to account for reduced levels of genetic variation in inland populations.

### Morphological analysis

**Figure 3:**
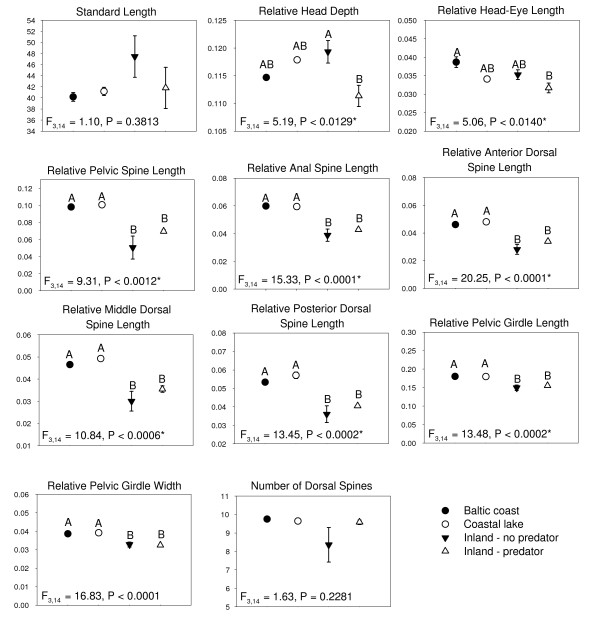
**Mean (± *SE*) for 10 morphological measurements and the number of dorsal spines arranged by group**. Standard length is reported in mm and relative measurements are in units per standard length. Whole model results of general linear mixed models (GLMM) between groups using population nested within group as a random factor are reported. Groups assigned different letters are significantly different (Tukey-Kramer HSD test). Asterix denote significant differences (α = 0.05)

Results of the analyses of the 11 morphological characters are summarized by population in Table 5 (See Methods for complete descriptions of morphological characters). Mean estimates of body length (SL) were highest for the inland populations without predators, but did not differ significantly from the other groups (Figure 3). Relative measures of head depth (per unit of standard length, SL^-1^) demonstrate that inland populations (with and without predators) have the most divergent morphology while coastal populations (Baltic coast, coastal lakes) show intermediate values for this trait (Figure 3). Relative head-eye length, on the other hand, show Baltic coast and inland lake without predator populations to have the most divergent morphology, with coastal lakes and inland lake - predator populations having intermediate values (Figure 3). Relative estimates of spine length (pelvic, anal, anterior dorsal, middle dorsal, posterior dorsal, girdle length and girdle width) consistently demonstrate significantly longer spines (SL^-1^) in coastal populations than in inland populations (Figure 3). The one measured meristic character, the number of dorsal spines, was variable in all populations with a mean of 9-10 spines (range 8-11) for all populations except BN, which had a mean of five spines (range 1-10) (Table 5). However, this character was not significantly different between groups (Figure 3).

**Table 5:** Population means for 10 morphological measurements (mm ± *SE*) and the number of dorsal spines (± *SE*)

Group	Site	Standard Length	Head Depth	Head-Eye Length	Pelvic Spine Length	Anal Spine Length	Anterior Dorsal Spine Length	Middle Dorsal Spine Length	Posterior Dorsal Spine Length	Pelvic Girdle Length	Pelvic Girdle Width	Number of Dorsal Spines
Baltic coast	BOV	43.1 ± 0.5	4.95 ± 0.06	1.48 ± 0.03	3.95 ± 0.09	2.42 ± 0.04	1.95 ± 0.05	2.01 ± 0.04	2.23 ± 0.04	7.76 ± 0.14	1.75 ± 0.04	9.87 ± 0.11
Baltic coast	BV	39.3 ± 0.7	4.50 ± 0.08	1.54 ± 0.02	3.85 ± 0.08	2.37 ± 0.05	1.74 ± 0.06	1.81 ± 0.06	2.06 ± 0.04	7.13 ± 0.10	1.48 ± 0.03	9.70 ± 0.13
Baltic coast	GB	40.8 ± 0.7	4.64 ± 0.08	1.72 ± 0.04	4.15 ± 0.10	2.45 ± 0.06	1.97 ± 0.05	1.91 ± 0.04	2.15 ± 0.06	7.34 ± 0.18	1.56 ± 0.05	9.87 ± 0.13
Baltic coast	LSV	39.2 ± 0.4	4.54 ± 0.07	1.60 ± 0.02	3.70 ± 0.08	2.20 ± 0.06	1.74 ± 0.04	1.71 ± 0.04	2.03 ± 0.05	6.95 ± 0.11	1.49 ± 0.02	9.60 ± 0.09
Baltic coast	TS	38.6 ± 0.6	4.38 ± 0.08	1.41 ± 0.04	3.99 ± 0.08	2.52 ± 0.06	1.84 ± 0.04	1.89 ± 0.04	2.20 ± 0.04	7.04 ± 0.17	1.50 ± 0.04	9.72 ± 0.14
Coastal lake	HV	40.7 ± 0.4	4.81 ± 0.06	1.40 ± 0.03	4.21 ± 0.08	2.52 ± 0.05	1.96 ± 0.04	2.02 ± 0.04	2.46 ± 0.04	7.22 ± 0.15	1.63 ± 0.03	9.47 ± 0.09
Coastal lake	KT	42.6 ± 0.4	5.01 ± 0.06	1.43 ± 0.04	4.25 ± 0.05	2.36 ± 0.04	2.03 ± 0.04	2.09 ± 0.04	2.37 ± 0.04	7.77 ± 0.10	1.60 ± 0.02	9.67 ± 0.12
Coastal lake	SB	40.2 ± 0.5	4.71 ± 0.07	1.38 ± 0.03	3.95 ± 0.07	2.45 ± 0.06	1.91 ± 0.03	1.96 ± 0.04	2.21 ± 0.04	7.24 ± 0.14	1.61 ± 0.03	9.80 ± 0.10
Inland - no predator	AT	53.8 ± 0.9	6.41 ± 0.10	1.82 ± 0.05	2.31 ± 0.08	1.62 ± 0.04	1.42 ± 0.05	1.53 ± 0.04	1.62 ± 0.04	7.83 ± 0.16	1.66 ± 0.03	9.13 ± 0.13
Inland - no predator	BN	58.8 ± 0.9	6.68 ± 0.11	1.81 ± 0.03	0.04 ± 0.04	1.68 ± 0.05	0.91 ± 0.11	0.81 ± 0.12	1.40 ± 0.04	7.30 ± 0.19	2.21 ± 0.05	4.63 ± 0.54
Inland - no predator	HST	43.8 ± 0.6	5.47 ± 0.09	1.57 ± 0.05	2.95 ± 0.04	1.77 ± 0.04	1.30 ± 0.03	1.42 ± 0.03	1.54 ± 0.03	6.40 ± 0.16	1.32 ± 0.03	9.57 ± 0.09
Inland - no predator	LT	39.6 ± 0.6	4.83 ± 0.07	1.51 ± 0.03	2.75 ± 0.06	1.64 ± 0.04	1.29 ± 0.03	1.39 ± 0.03	1.60 ± 0.04	6.45 ± 0.13	1.27 ± 0.02	9.30 ± 0.13
Inland - no predator	LN	41.4 ± 1.2	4.80 ± 0.16	1.56 ± 0.05	2.95 ± 0.10	2.20 ± 0.06	1.49 ± 0.05	1.66 ± 0.05	2.06 ± 0.04	6.89 ± 0.20	1.37 ± 0.04	9.14 ± 0.11
Inland - predator	VST	40.8 ± 0.9	4.39 ± 0.10	1.28 ± 0.04	3.04 ± 0.10	1.79 ± 0.05	1.40 ± 0.03	1.53 ± 0.04	1.66 ± 0.03	6.18 ± 0.13	1.34 ± 0.03	9.65 ± 0.12
Inland - predator	DT	44.8 ± 0.5	5.28 ± 0.09	1.53 ± 0.03	3.12 ± 0.06	1.87 ± 0.04	1.54 ± 0.03	1.63 ± 0.02	1.83 ± 0.03	7.14 ± 0.08	1.48 ± 0.03	9.27 ± 0.12
Inland - predator	HM	40.3 ± 1.1	4.37 ± 0.09	1.15 ± 0.03	2.60 ± 0.06	1.64 ± 0.05	1.26 ± 0.04	1.28 ± 0.04	1.53 ± 0.04	6.22 ± 0.19	1.33 ± 0.03	9.88 ± 0.08
Inland - predator	RT	30.1 ± 0.4	3.25 ± 0.05	0.87 ± 0.03	1.99 ± 0.05	1.37 ± 0.04	0.98 ± 0.03	0.97 ± 0.02	1.25 ± 0.03	4.60 ± 0.09	0.98 ± 0.02	9.77 ± 0.12
Inland - predator	SKT	53.1 ± 0.5	6.03 ± 0.11	1.88 ± 0.04	3.96 ± 0.06	2.23 ± 0.03	1.92 ± 0.03	2.07 ± 0.03	2.16 ± 0.02	8.60 ± 0.14	1.69 ± 0.03	9.33 ± 0.10

See Table 1 for description of site abbreviations and text for measurement descriptions.

A cluster analysis based on population means across all 11 morphological characters demonstrated strikingly similar morphology between Baltic coast and coastal lake populations (Figure 2b). Inland populations showed greater divergence in morphology than coastal populations (Figure 2b). Although there is some morphological clustering among inland populations that share similar predation regimes, there is also clustering of populations that have disparate predation regimes, suggesting that overall phenotypic appearance may evolve independently in these populations (Figure 2b).

Results of the principal components analysis (PCA) showed that the first two principal components had Eigenvalues greater than one and explained a combined 80.6% of the total variation of the full data. Therefore only these two axes were used in subsequent analyses. Principal components axis 1 (PC1) represented 47.7% of the variance and had high loading scores (> 0.40) for pelvic spine length, anal spine length, and all dorsal spine lengths after varimax rotation (Table 6). Principal components axis 2 (PC2) represented 32.9% of the variance and had high loading scores for standard length, head depth, head length, and pelvic girdle width (Table 6). Therefore, PC1 corresponds to a "spine length" function while PC2 corresponds to a "body size" function. Only pelvic girdle length had high loading scores for both PC1 and PC2 (Table 6).

**Table 6:** Principle components analysis of loading scores for 10 morphological characters

	PC1	PC2
Standard Length	-0.08	0.95
Head Depth	0.14	0.79
Head-Eye Length	-0.11	0.93
Pelvic Spine Length	0.90	-0.17
Anal Spine Length	0.85	0.12
Anterior Dorsal Spine Length	0.92	0.10
Middle Dorsal Spine Length	0.92	0.11
Posterior Dorsal Spine Length	0.91	0.13
Pelvic Girdle Length	0.49	0.74
Pelvic Girdle Width	0.13	0.83

Proportion of variation explained	47.7	32.9

Proportion of variation explained by each axis is shown. See text and Figure 6 for measurement descriptions.

**Figure 4:**
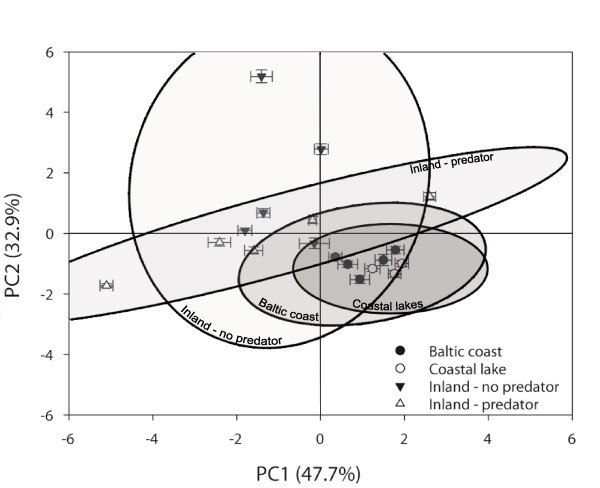
**Principal components scores (mean ± *SE*) for individual populations arranged by group**. 95% confidence density ellipses are drawn for all individuals within a group.

Visualization of the PCA results reveals tight clustering of Baltic coast and coastal lakes in PC space (Figure 4), confirming their morphological similarity. Inland populations, on the other hand, showed much greater variation than coastal populations. Inland populations without predators showed greater variation in PC2 scores than in PC1 scores and the opposite is true for inland predator populations (Figure 3). Thus, the introduction of predators in inland populations appears to have reduced variation in body size, accompanied by an increase in the variability of spine length (Figure 4).

To test whether variation in PC scores differs significantly between groups and populations nested within groups, we constructed a nested multivariate analysis of variance (MANOVA) on PC1 and PC2. Our results show strong support for morphological divergence among groups and significant differences between populations nested within groups (Table 7).

**Table 7:** Nested MANOVA of PCA scores testing the difference between groups and population nested within group (random effect)

	Wilk's lambda	DF	~F	P
All groups				
Whole model	0.0330	34.1030	122.3634	< 0.0001
Group	0.1528	6.1030	267.4450	< 0.0001
Population [Group]	0.0797	28.1030	93.5370	< 0.0001

### *P*_ST _- *F*_ST _comparisons within groups

**Figure 5:**
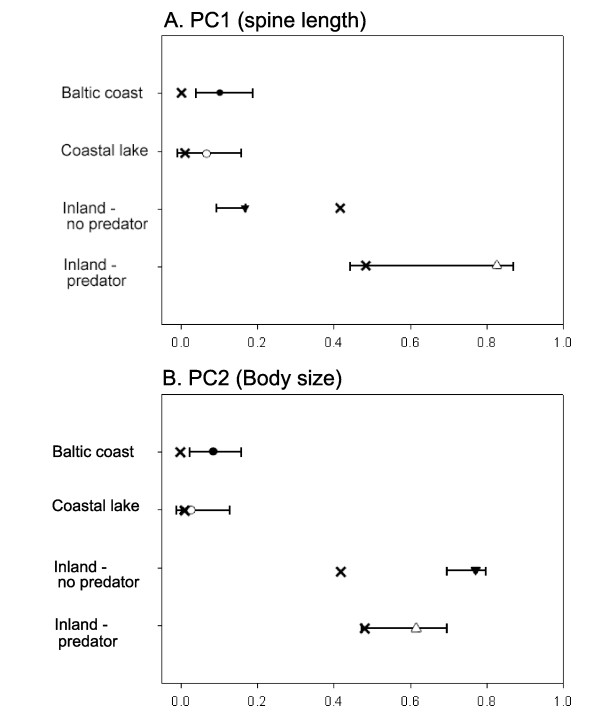
**Within-group *P*_ST _- *F*_ST _comparisons for A) PC1 and B) PC2**. Global *F*_ST _are shown as an X. Mean *P*_ST _estimates (± 95%CI) are shown as symbols.

Within-group estimates of neutral genetic divergence (*F*_ST_) and phenotypic divergence (*P*_ST_) in Baltic coast and coastal lake groups demonstrated low estimates for each measure (Table 4, Figure 5). The Baltic coast group had *P*_ST _>*F*_ST _with non-overlapping 95% confidence intervals for both PC1 and PC2 suggesting that divergent selection on both spine length and body size is occurring in populations of this group. Coastal lake *P*_ST _estimates, on the other hand, had overlapping 95% confidence intervals with *F*_ST _(Table 4, Figure 5) which suggests that within this group, one cannot distinguish between the effects of natural selection and genetic drift on phenotypic appearance. Within the inland no predator group, we found *P_ST _*<*F*_ST _for PC1 indicating stabilizing selection on spine length and the opposite trend, namely *P*_ST _>*F*_ST _for PC2, suggesting divergent selection in body size in this group (Table 4, Figure 5). Finally, inland populations with predators show *P*_ST _>*F*_ST _for PC1 and PC2 suggesting divergent selection on spine length and body size, but estimates of *P*_ST _are more variable than any other estimate and confidence intervals overlap *F*_ST _values, making a judgement as to whether natural selection or genetic drift is operating equivocal (Table 4, Figure 5).

To test whether our measures of *P*_ST _are a robust and fair measure of *Q*_ST_, we investigated the estimate of the critical *c/h*^2 ^ratio [27] for both PC1 and PC2 in cases where *P*_ST _>*F*_ST _and the inverse relationship (i.e. *h*^2^/*c *ratio) when *P*_ST _<*F*_ST_. Low values of these ratios indicate more robust support for *P*_ST _to approximate *Q*_ST _in their various scenarios [27]. If we assume that body size is moderately heritable so that *h*^2 ^= 0.4 (see Discussion), and because *c *cannot exceed unity, using the lower 95% confidence limit for *P*_ST _in cases where *P*_ST _>*F*_ST _yields low values of *c/h*^2 ^critical values of 0.200 and 0.287 for Baltic coast populations PC1 and PC2 respectively (Table 4). This implies that a 70-80% lower *c *than *h*^2 ^would be necessary to attribute the observed phenotypic divergence to drift. Thus, we may interpret our results to be robust under this scenario to the null expectation. Similarly, a low *c/h*^2 ^critical value of 0.125 was found in inland no predator group for PC2 also demonstrating a robust signature of divergent selection within this group. Under the one instance where *P*_ST _<*F*_ST _in inland no predator PCI, the *h*^2^/*c *ratio was low at 0.283 and we may conclude that *Q_ST _*<*F_ST_*, i.e. a signal of convergent evolution/stabilizing selection on spine length in inland populations without predators.

## Discussion

In this study, we used molecular and morphological data to quantify differentiation between populations from coastal areas of the Baltic Sea and inland populations that have been isolated since the last deglaciation. In general, we find that Baltic coast and coastal lake populations are similar genetically and morphologically. Inland populations, on the other hand, show greater genetic and morphological divergence. The recent introduction of predators into some inland populations also appears to have altered the evolution of body shape and spine length as inland populations with predators varied considerably in spine length but showed much greater conservatism in body size than inland populations without predators. Taken together, these results suggest that nine-spined stickleback populations in northern Sweden are strongly influenced by a combination of recent glacial activity, isolation/drift, and natural selection.

### Genetic divergence

Our results support the hypothesis that inland populations of nine-spined sticklebacks were isolated from coastal populations following the recent deglaciation of the Baltic Sea. Our first line of support for this hypothesis comes from the physical location of these sites which were among the first areas to be exposed after the retreat of the Scandinavian Ice Sheet circa 11 - 10 k cal. ybp [22,23]. A plausible scenario is that these populations originated from Baltic stock and were isolated as the land uplifted from glacial rebound forming lakes and ponds [24,25]. Additional support for this hypothesis is venerated by the observation that few natural populations of nine-spined sticklebacks are found in northern Sweden above or below the highest coastline exposed after the last glacial maximum [28].

In agreement with the hypothesis for isolation of inland populations, we found lower levels of genetic differentiation, as measured by Nei's *D*_A_, *F*_ST _and standardized *F*_ST_, in coastal sites and very high levels of genetic differentiation including the fixation of different alleles in many inland populations. Accordingly, we found higher levels of genetic variation as measured by *H*_O_, π, and *h*_s _in coastal populations than in inland populations. These last two findings are consistent with a pattern of isolation and genetic differentiation of populations through genetic drift and/or founder effects. Based on our results, these decreases in *H*_O _do not appear to be due to a recent bottleneck in inland populations, with the exception of VST where significant results of simulated genetic bottlenecking were detected. We also found that populations from coastal lakes appear to be genetically indistinguishable from those emanating from the Baltic Sea, suggesting a separation so recent that genetic differences have yet to become apparent. These results strongly suggest that coastal populations are closely related whereas inland populations have existed in isolation for quite some time, and that genetic drift is responsible for the divergence in allelic frequencies and fixation of certain alleles.

Our support for a reduction in genetic variability among inland populations echoes findings in freshwater populations of the closely related three-spined stickleback (*Gasterosteus aculeatus*) that generally have reduced genetic variability [29-31] as a result of isolation from presumably ancestral anadromous populations [32]. Similar models for post-glacial colonization by nine-spined sticklebacks have also been proposed and confirmed for North American populations [8,33]. A recent study of nine-spined sticklebacks by Shikano and colleagues demonstrated similar patterns of isolation from anadromous ancestral populations of this species in Europe [9]. In their study, coastal populations from the Baltic sea had high levels of genetic variation as measured by allelic richness and heterozygosity when compared to populations from freshwater systems near the Baltic while higher levels of genetic differentiation, as measured by *F*_ST _and *D*_A_, were prevalent in freshwater but not in coastal populations [9]. The authors concluded that these patterns of genetic variation and genetic differentiation are consistent with postglacial recolonization of freshwater habitats, and subsequent isolation reducing variation in these populations through genetic drift and founder effects. Since Shikano and colleagues' study encompassed sampling from a much larger geographic area than studied here, it appears that this pattern of isolation is common in the Baltic region and that recent glacial history has greatly affected the current distribution of these fishes.

### Morphological divergence

Our results show many morphological similarities between Baltic coast and coastal lake populations of nine-spined sticklebacks, mirroring the pattern of low levels of genetic differentiation. Given that coastal lakes have been isolated from the Baltic due to land uplift over a relatively short period of time (< 100 yrs), it is not surprising that there has been little differentiation in morphology compared to the relatively greater morphological divergence in inland populations. Moreover, piscivorous fish such as pike and perch are not currently detected in two of the three coastal lakes [28] but were likely present in these habitats prior to and during lake formation. Therefore morphological divergence in the absence of predation may be expected in these populations in the future, but perhaps not over the short time since isolation from the Baltic.

Inland populations of nine-spined sticklebacks displayed higher morphological diversity as compared to coastal populations. We also found evidence of morphological differences in both body size and spine length with respect to the presence of fish predators. Additionally, we found highly divergent morphological variation in some inland populations. For example, the populations with the two most divergent morphologies, BN and RT, show the greatest differences in mean body size. The BN population also has a reduction in the number of dorsal spines and dorsal spine length, and all but one individual completely lacked pelvic spines. This pattern of pelvic spine loss and an overall reduction in spines has been demonstrated in several species of stickleback [16,19,34] and may be due to an ecological escape from predation pressure, an ion deficiency related to calcification and bone deposition and/or increased invertebrate predation pressure in these populations [16,20,35].

Nine-spined sticklebacks that exist in ponds where they are the only fish species can obtain much larger body sizes than their coastal counterparts, presumably because of the absence of fish predation combined with interspecific competition for resources and/or fecundity selection [11,12]. Confirming this prediction, we observed a trend for individuals hailing from inland populations without predators to be larger, on average, than both Baltic populations and inland lakes with predators. A similar pattern of larger size encountered in populations that either lack predators or have non-gape limited predators has been shown in three-spined sticklebacks [36] and brook sticklebacks (*Culaea inconstans*) [37], strongly suggesting that predation limits body size in this and other species of sticklebacks.

Despite having a smaller range in body size than their counterparts that hail from predation-free lakes, nine-spined sticklebacks from predator lakes have much greater variation in spine length. Although spine lengths are significantly smaller in inland populations compared to coastal populations, there is a trend for inland populations with predators to show longer spines (per unit of body length), on average, than inland populations without predators. The implication, therefore, is that spine length has increased in these populations as a result of the recent introduction of predatory fish. However the great variation in spine length exhibited among these populations within this group suggest that these populations may be responding to predation pressure differently. This great variation spine length could be caused by differing predation regimes within these lakes due to differences in predator communities, population densities of predators, differences in the relative exposure time to predators, and/or the recent extinction of predators within at least one lake. Although our results strongly suggest a role of predation, we cannot rule out that other ecological factors not measured in this study such as water chemistry may affect morphological variability in predator lakes.

### *P*_ST _- *F*_ST _comparisons

Quantitative comparisons between morphological and neutral genetic divergence as estimated by *Q*_ST _- *F*_ST_, have been used as metric to investigate the potential for natural selection to influence morphological variation in many populations or species [38,39]. The analogous comparison based solely on phenotypic data (i.e., *P*_ST _- *F*_ST_) has been highly criticized, particularly because it is difficult to tease apart the variance in phenotype attributable to environmental or genetic effects in wild populations. Thus, some advocate that *Q*_ST _- *F*_ST _comparisons should only be performed under controlled conditions in common garden experiments [40]. While such approaches are preferable, we argue that there can be some value to *P*_ST _- *F*_ST _comparisons in natural populations. For example, in a recent meta-analysis that compared estimates of *Q*_ST _- *F*_ST _from different types of studies, estimates of *P*_ST _from wild populations do not yield higher estimates than studies that use either broad or narrow sense estimates of additive genetic variation [39]. Secondly, studies of *P*_ST _in wild populations show meaningful variance among populations where a common garden approach may not be easily applied [27,29,41]. For example, in order to quantify additive genetic variation in our study of 18 natural populations with all potential crosses and multiple family groups taken to the *F*_2 _generation would be a feat of herculean proportions in terms of time, scale and expense. Finally, in light of the criticism of *P*_ST _as a substitute for *Q*_ST_, it should also be kept in mind that common garden estimates of *Q*_ST _may be inappropriate to compare to *F*_ST _because the genetic basis of the phenotypes on which selection may potentially act may be partly genetic but non-additive (i.e. epigenetic), or environment-dependent. Thus we believe that *P*_ST _- *F*_ST _studies in natural populations do have some merit although we advocate caution in its interpretation.

Acknowledging the aforementioned concerns, we compared *P*_ST _estimates for both spine length (PC1) and body size (PC2) within groups to estimate the relative influence of natural selection and genetic drift on the evolution of morphological phenotypic variation within our groups of interest. Despite the great morphological similarity within the Baltic coast group, it demonstrated *P*_ST _>*F*_ST _in both spine length and body size. Given the high gene flow experienced within this group, the most likely explanation is that these slight morphological differences could be explained by phenotypic plasticity in response to local environmental variation. However it should be noted that our *F*_ST _estimates are so low that any variation in morphology would likely be greater than genetic divergence and so this result should be viewed cautiously. In coastal lakes, *P*_ST _≈ *F*_ST _for these estimates making distinctions between selection and genetic drift equivocal. In inland lakes without predators, we found a robust *F*_ST _- *P*_ST _pattern of convergent evolution and stabilizing selection on reduced spine length strongly suggesting that reduced spine length is advantageous in single species lakes, potentially to aid escape of fish from invertebrate predators. A robust pattern of divergent selection and local adaptation on body size is also evident in inland no-predator lakes. Finally, great variation in body size and spine length within the inland predator group may also be indicative of divergent selection on these traits in these populations but confidence intervals are wide making a realistic determination difficult. Taken together, these results show opposite and robust patterns of convergent versus divergent selection on spine length based on the presence or absence of predation, strongly implicating the recent exposure to predators as a significant factor shaping phenotypic differences between these populations.

The degree to which our main focal phenotypic characters, body size and spine length, differ in their phenotypic response to selection is not currently known in nine-spined stickleback but may vary among different populations due to different environment conditions and standing levels of genetic variation [3,5]. Studies on other fish species show that fish predators potentially can induce phenotypic changes in body shape and morphology without necessarily changing the background genetic structure [42-44]. For example, one study showed that cues of a predatory fish induced a deeper body and longer dorsal spines in a sunfish (*Lepomis gibbosus*) [43]. The genetic component of the traits studied here remain unknown as previous studies investigating genetic differences among populations of nine-spined stickleback using a common-garden approach have not yet reported heritablities for these traits [13,45]. Among three-spined sticklebacks, morphological traits of predator-naive and predator-sympatric populations demonstrate high values of heritability for body shape (*h*^2 ^= 0.67, 0.94) and length (*h*^2 ^= 0.92, 0.82) and moderate values for relative spine length (*h*^2 ^= 0.34, 0.39) [46]. Other studies of three-spined sticklebacks have shown that body size is moderately heritable (*h*^2 ^= 0.42) [47] or that heritability may vary from negligible (*h*^2 ^= 0.007) to moderate (*h*^2 ^= 0.313) due to environmental factors such as rearing salinity [5]. Taken together, these results suggest that in most cases morphological traits such as body size and spine length are moderately to highly heritable in three-spined sticklebacks, but that the ratio between genetic and environmental variance (and hence the heritability) may not be equal across populations.

## Conclusions

Understanding the causes underlying morphological differences between populations is a fundamental question in evolutionary biology. Our power to explore differences in morphology among inland populations with disparate predation regimes is significantly enhanced by our knowledge of predator stocking and 'fisher knowledge' based on over 100 years of data collection within inland lakes. Our two findings that 1) fish hailing from inland no predator lakes appear to be convergent selection on small spine length and divergent selection on body size and 2) fish from predator lakes have a highly variable response in spine length and body size strongly suggests that the recent introduction of salmonids in these lakes have influenced morphology in nine-spined sticklebacks in these populations. However, our current study cannot determine whether additional factors such as water chemistry [20,35] and invertebrate predation [18] can contribute to phenotypic variation in our populations of stickleback. Additionally, the amount of environmental variation and heritability of phenotypic traits within and between populations is outside of the scope of this study but certainly warrant additional investigation along these lines. This research also highlights the importance of studying morphological and genetic variation on a fine ecological scale in order to determine the environmental factors responsible for shaping the shared and unique features of evolutionary histories.

## Methods

### Study area

Field sampling of nine-spined stickleback populations occurred during May - August 2007 and June 2008 in the counties of Västerbotten and Västernorrland, northern Sweden (Table 1, Figure 1). In total, 18 locations were visited including eight populations from the Baltic Sea coast consisting of five populations from coastal bays (Baltic coast) and three populations from lakes situated within a km of the Baltic Sea (coastal lakes). An additional 10 populations were sampled from inland lakes and ponds that have been isolated from the Baltic Sea for more than 9000 cal. ybp (Figure 1). Of the 10 inland lakes and ponds, five are single species lakes where nine-spine sticklebacks are the only fish species recorded (inland - no predators, Table 1). Stickleback populations in the remaining five inland lakes and ponds have a known history of predation (inland - predators). Predators such as brown trout (*Salmo trutta*) and rainbow trout (*Oncorhynchus mykiss*) have been stocked in these lakes for recreational angling purposes. Records show that these lakes (except HM) have been stocked as early as 1959 and many have been stocked extensively in the 1980s and 1990s (Table 1). No records of the presence of these fish precede the known stocking dates. Records indicate that one of our sampling sites (SKT) was stocked with brown and rainbow trout in 1985, but surveys after 2000 indicate that these introduced species are no longer present in this lake. Nevertheless, this lake was classified as a predator lake based on the very recent exposure to predation.

The selection of locations was based on information in the PIKE Database [28]. The database contains records of fish community composition and introductions for more than 19,000 Swedish lakes. Information about fish species is based on interviews with local fishermen and surveys using a minimum of eight minnow traps and/or four multi-mesh gill net sets (bottom set, monofilament nylon and measured 30 × 1.5 m with mesh size ranging between 5 and 55 mm) [48].

### Fish collection and morphological measurements

**Figure 6:**
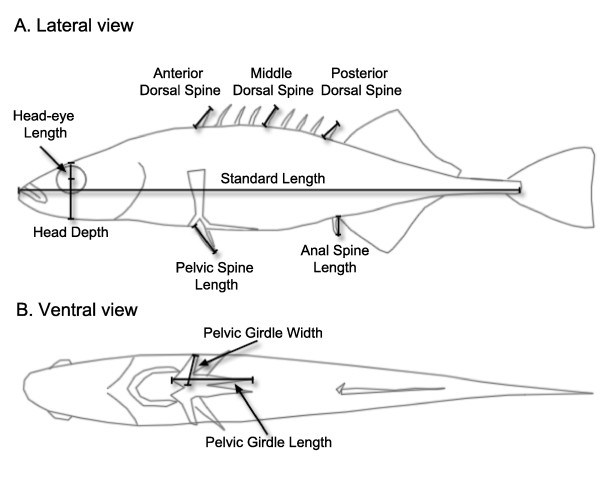
**Morphological traits measured for this study. See text for detailed description of measurements**.

Adult nine-spined sticklebacks were caught using baited minnow traps and landing nets. Fish were immediately placed on ice and transported to the laboratory for genetic and morphological analyses. There, specimens were defrosted and morphological traits were measured with a digital calliper. Traits measured included standard length (SL, tip of snout to tip of caudal peduncle), head depth (top of head to bottom of head through centre of eye perpendicular to SL), head-eye length (top of head to centre of eye perpendicular to SL), length of pelvic spine, length of anal spine, and length of anterior, middle and posterior dorsal spine (Figure 6a). The total number of dorsal spines was also counted. For individuals with even numbers of dorsal spines, middle dorsal spine length was an average of the length of the two middle spines. After measurements were taken, the right pelvic fin (or the caudal fin in the BN population) was clipped for genetic analyses, and individual fish were stained in order to measure the pelvic girdle.

To prepare the staining of the pelvic girdle, fish were first fixed according to the following scheme: 95% EtOH for 24 h, 70% EtOH for 24 h, 50% EtOH for 24 h, 20% EtOH for 24 h and 24 h in distilled H_2_O. Fish were then transferred to a 0.4 g/l aqueous solution of KOH containing 0.425 g of Alizarin red to stain bony parts. Fish were kept in the solution for 3 h and were then transferred to 0.4 g/l aqueous solution of KOH without dye. After 24 h, fish were placed in 50% iso-propanol for long-term storage. After staining, the length and width of the pelvic girdle was measured using callipers (Figure 6b).

### Microsatellite analysis

Total genomic DNA was extracted from finclips of all individuals in each sampled location using a DNeasy ^® ^Blood & Tissue Kit (product #69581, Quigen, Valencia, USA). Nine polymorphic microsatellite markers (*Pbbe1125, Stn19, Stn49, Stn96, Stn148, Stn163, Stn173, Stn196, Stn198*) previously used to characterize *P. pungitius *[49] were individually labelled with florescent primers. Microsatellite markers were amplified using polymerase chain reaction (PCR) in a 10 μl reaction using a multiplex PCR kit (Qiagen Inc.). PCR conditions for each reaction using the multiplex kit were 5 μl of Qiagen Master Mix (containing HotStarTaq DNA polymerase, dNTPs and 3 mM MgCl_2_), 0.625 μM of each primer, and 3 μl of genomic DNA. Temperature profile for thermal cycling: 96°C for 15 min followed by 35 cycles of 94°C for 30 sec, 60°C for 90 sec, 72°C for 90 sec, and a final 20 min extension at 72°C. Fragment lengths of PCR products were analyzed using CEQ™ 800 Genetic Analysis System (Beckman Coulter Inc., Brea, USA).

Microsatellite data were analyzed with ARLEQUIN v. 3.11 [50] to test for Hardy-Weinberg equilibrium (Fisher's exact test) and for genotypic disequilibrium for pairs of loci within populations (Fisher's exact test). Loci were checked the scoring errors and the presence of null alleles using MICROCHECKER v 2.2.3 [51]. We also tested all loci for neutrality using the program LOSITAN using a multi-step mutation model and 10,000 replicates of data collection [52,53].

A single microsatellite locus, *Stn49 *showed a mutation consisting of a one base pair insertion. This mutation is common in eastern European lineages of *P. pungitius *[9] and it was included in all subsequent analyses except for analyses conducted with MICROCHECKER, LOSITAN, and BOTTLENECK because this locus does not follow a typical dinucleotide stepwise mutation pattern [9].

Nei's genetic distance (*D*_A_) [54] was used to construct a phylogeny based on microsatellite data for the 18 nine-spined stickleback populations using the program POPULATIONS v. 1.2.30 [55]. Levels of bootstrap support were obtained from 10,000 replicates and the resulting neighbour-joining tree was drawn with POPULATIONS.

Genetic differentiation, as measured by *F*_ST _[56], was calculated between each population pair with ARLEQUIN. Significance of global pairwise *F*_ST _values was estimated with ARLEQUIN using 10,000 permutations. In order to compare measures of genetic differentiation corrected for differences between populations with different levels of allelic richness and heterozygosity, we calculated standardized *F*_ST _values with GENODIVE v. 2.0b18 [57].

We tested for differences in within-group genetic variation (Baltic coast, coastal lakes, inland lakes with or without predators) and between coastal (pooled Baltic coast, coastal lakes) and inland lakes (pooled predators, no predators). Our estimates of genetic variation included observed heterozygosity (*H*_o_), expected heterozygosity (*H*_e_) allelic richness (*A*), and global *F*_ST _values with FSTAT v 2.9.3.2 [58] using the "comparison among groups of samples" subfunction. Significance was ascertained for these parameters over 10,000 permutations.

To investigate whether populations bear the signature of a recent fluctuation in population size, we investigated heterozygosity deficiency and heterozygosity excess with respect to gene diversity using the program BOTTLENECK v. 1.2.02 with 1,000 replications of a two-phase mutation model [59]. The two-phase model of mutation consisted of mostly of one-step changes with a low percentage of multistep changes (9:1), recommended by Di Rienzo *et al*. [60] for microsatellites.

### Morphological analysis

We first tested for differences between groups (Baltic coast, coastal lakes, inland no predators, inland predators) using a general linear mixed model (GLMM) using group as a fixed factor and population nested within group as a random factor to ascertain whether groups significantly differed in standard length and the number of dorsal spines. All remaining morphological measurements were first divided by standard length to obtain a relative morphological measurement per unit of standard length (SL^-1^) prior to GLMM analysis. A Tukey-Kramer honestly significant difference *post-hoc *analysis was conducted to determine which groups gave rise to significant differences.

A morphological cluster analysis was conducted on population means of all 11 morphological characters including the number of dorsal spines. Principal components analysis (PCA) was performed on 10 morphological characters (excluding the meristic character) and a varimax rotation [61] was applied to all components that had Eigenvalues greater than 1. A nested multivariate analysis of variance (MANOVA) was then used to test for differences in morphological PC axes between all groups, with populations nested within groups. Since many individuals in the BN population lacked dorsal, pelvic and anal spines or had highly reduced spine lengths and therefore may have contributed a disproportionate amount of variation to the PCA, we constructed the PCA and all subsequent analyses with and without the BN population. However, all significant differences in MANOVAs between groups were detected irrespective of whether BN was included, so we present only the results with BN included.

### Calculation of *P*_ST_

In can be shown that (for diploid species, assuming purely additive gene action) the quantity

$$Q_{ST}=\frac{V_{A,b}}{V_{A,b}+2V_{A,w}}$$

(where *V_A, b _*and *V_A, w _*are the morphological additive genetic variance components between and within populations) is expected to take the same value as *F*_ST _calculated from the genes that determine the trait [62,63]. Hence, if *F*_ST _is calculated from neutral markers such as the microsatellites we analyzed, then for a quantitative trait under divergent selection *Q*_ST _>*F*_ST _and under stabilizing or convergent selection *Q*_ST _<*F*_ST_. Unfortunately, the common garden experiments needed to estimate the additive genetic variance components of a character are difficult to realize for many species. Therefore, *Q*_ST _is often approximated by *P*_ST_, which is calculated directly from the total phenotypic (i.e. combined genetic and environmental) variance components [64]:

$$P_{ST}=\frac{V_{b}}{V_{b}+2V_{w}}$$

Obviously, how well *P*_ST _approximates *Q*_ST _depends on whether the total phenotypic variance within and between populations reflects the additive genetic variance. The heritability *h*^2 ^relates the additive variance within a population to the total variance: *V_A, w _*= *h*^2 ^*V_w_*. We can similarly envision a "population level heritability" *c *so that *V_A, b _*= *c V_b_*. If we are willing to make the simplifying assumptions that both *h*^2 ^and *c *are equal for all populations, then [27,63]:

$$Q_{ST}=\frac{cV_{b}}{cV_{b}+2h^{2}V_{w}}$$

Algebraic rearrangement then yields the relation between *Q*_ST _and *P*_ST _expressed as a simple function of the ratio *c/h*^2^:

$$Q_{ST}=\frac{P_{ST}}{P_{ST}+{\left(\frac{c}{h^{2}}\right)}^{-1}\left(1-P_{ST}\right)}$$

Thus, we can calculate *P*_ST _and evaluate which value the ratio *c/h*^2^should take for *Q*_ST _to equal *F*_ST_, that is:

$$\frac{c}{h^{2}}=\frac{F_{ST}\left(P_{ST}-1\right)}{P_{ST}\left(F_{ST}-1\right)}$$

In other words, if for some trait we find that *P*_ST _<*F*_ST_, we can calculate which ratio *c/h*^2 ^would generate this result when in fact *Q*_ST _= *F*_ST_. In the reverse situation when *P*_ST _>*F*_ST_, we can take the inverse of the *c/h*^2 ^ratio (i.e., *h*^2^/*c*) to test the robustness of our estimates. If the required ratio is extreme, i.e. *c *< <*h*^2 ^we may be confident that the conclusion based on a *P*_ST _- *F*_ST _comparison would not be altered if we had estimated *Q*_ST _directly. Therefore, we report "uncorrected" values of *P*_ST _in the results, and use the above formula when interpreting the robustness of our results.

To calculate *P_ST_*, we partitioned phenotypic variance in within- and between population components by ANOVA (assuming unequal variances) in Matlab (version 7.5, MathWorks, Natick, Massachusetts, USA). We then calculated the within-group mean pairwise *P*_ST _of all pairs of populations. If there are *n *populations in the group, there are *n *(n-1)/2 pairs of populations. For example, for the *n *= 5 coastal bay populations, *P*_ST _is calculated as the mean of 10 pairs of populations. For between-group *P*_ST _we calculated the mean pairwise *P*_ST _of all pairs of populations where each pair consists of a population from the one group and a population from the other group. If there are *n*_1 _populations in the one group and *n*_2 _populations in the other, there are *n*_1 _*n*_2 _between-group pairs. Because each population is part of several pairwise comparisons in the calculation of *P*_ST_, we used bootstrapping to calculate 95% confidence intervals.

Unless otherwise noted, all statistical tests were performed with JMP IN™ statistical software package version 7.0 (SAS Institute Inc. Cary, North Carolina, USA). Means are reported throughout text as ± one standard error of the mean (*SE*).

## Authors' contributions

KBM conducted all molecular and morphological analyses and drafted the manuscript. DL collected samples and measured morphological traits. FJ and GE organized collections and supervised morphological analyses. FB conducted *P*_ST _calculations and participated in the design and coordination of the study. All authors read and approved the final manuscript.
